# Auditory steady state response in pediatric audiology

**DOI:** 10.1590/S1808-86942010000600010

**Published:** 2015-10-19

**Authors:** Ana Emilia Linares, Orozimbo Alves Costa Filho, Maria Angelina Nardi de Souza Martinez

**Affiliations:** 1 PhD-FMUSP, Speech and Hearing therapy - Associação dos Pais e Amigos dos Deficientes Auditivos de Sorocaba; 2 Full Professor - University of São Paulo at Bauru; 3 PhD; Professor - PUC-São Paulo and Clinical Director - Associação dos Pais e Amigos dos Deficientes Auditivos de Sorocaba

**Keywords:** hearing, electrophysiology, hearing loss

## Abstract

The main issue regarding pediatric audiology diagnosis is determining procedures to configure reliable results which can be used to predict frequency-specific hearing thresholds.

**Aim:**

To investigate the correlation between auditory steady-state response (ASSR) with other tests in children with sensorineural hearing loss.

**Methods:**

Prospective cross-sectional contemporary cohort study. Twenty-three children (ages 1 to 7; mean, 3 years old) were submitted to ASSR, behavioral audiometry, click audiometry brain stem response (ABR), tone burst ABR, and predicting hearing level from the acoustic reflex.

**Results:**

the correlation between behavioral thresholds and ASSR was (0.70- 0.93), for the ABR tone burst it was (0.73 -0.93), for the ABR click it was (0.83-0.89) only at 2k and 4 kHz. The match between the ASSR and the hearing threshold prediction rule was considered moderate.

**Conclusion:**

there was a significant correlation between the ASSR and audiometry, as well as between ABR click (2k and 4 kHz) and for the ABR tone burst. The acoustic reflex can be used to add information to diagnosis in children.

## INTRODUCTION

The attempt to early detect and identify hearing impairment is associated with the need for proper intervention, providing the child with conditions to develop speech, language, hearing and also the social, psyche and educational aspects.

With the Neonatal Auditory Screening programs (NAS), together with the Auditory Health programs, we saw the possibility of early diagnosis and treatment. However, the diagnostic process can only be considered complete when one specifically identifies the type, degree and configuration of this population's hearing loss^1^, ^2^, ^3^.

The Brainstem Auditory Evoked Potential (BAEP) with the click and tone burst stimuli, otoacoustic emissions, Immittance values and Visual Reinforcement Audiometry (VRA) and, today, the most promising method for hearing assessment: auditory steady state brainstem audiometry (ASSBA), are all part of the set of tests (electrophysiological, electroacoustic and behavioral) to which the child is submitted for audiological investigation.

The electrophysiological measurement is the most employed tool to identify and characterize the hearing loss in the population of infants and children without cognitive and motor conditions to undergo VRA, or those who do not provide reliable responses during the test.

The main issue surrounding the pediatric audiological diagnosis is to establish the procedures which provide reliable and objective results, which can be used in the prediction of hearing thresholds by specific frequency. Thus, these thresholds are applicable in the prescription of the technological characteristics of amplification, fostering the auditory and language development of children.

This study aimed at correlating the ASSBA with the click and tone burst BAEP results, tonal audiometry (VRA), as well as correlating the degree of hearing loss indicated in the ASSBA as the one suggested by the hearing threshold prediction rule based on the acoustic reflex in children with different degrees of sensorineural hearing loss.

## MATERIALS AND METHODS

The present study (research protocol # 0492/07) was submitted to and approved by the Ethics in Research Committee of Project analysis.

The sample was made up of 23 children with bilateral sensorineural hearing loss, aged between 1 and 7 years, of both genders.

Inclusion criteria included bilateral sensorineural hearing loss, of consistent and reliable responses upon audiometry (VRA or ludic), as well as lack of neurological dysfunction and proper middle ear function.

The equipment used was the AZ7 Middle Ear Analyzer from - *Interacoustics, Interacoustic* (AC33) audiometer with in-the-ear phone, supra-aural and bone vibrator. The audiometry was carried out in a broad acoustic room. In order to do the BAEP with click, tone burst and ASSBA we used the *SmartEP - Intelligent Hearing Systems (Auditory Evoked Potencials System)*:

The procedures done were: otoscopy and tympanometry as acoustic admittance (Ya), with a 226 Hz probe frequency. In order to investigate the contralateral acoustic reflexes we employed 0.5k; 1k; 2k; 4kHz stimuli and broad band noise (white noise), recorded with the conventional probe at 226 Hz dBHL.

In order to study the behavioral auditory threshold prediction based on the acoustic reflex we used the auditory threshold prediction rule^4^. All the thresholds obtained in dBHL were converted to dBSPL, with the aim of employing them in the auditory threshold prediction rule. The Immittance metering device was physiologically calibrated as per suggested by the author^5^.

In the Visual Reinforcement Audiometry we used in-the-ear phones^6^, ^7^.

Ludic tonal audiometry was carried out after two years of age with in-the-ear phones; it was necessary to condition the child in order to perform the ludic tasks (fitting), each time he/she perceived the sound stimulus.

The BAEP (Click) was initially carried out in the intensity of 80 dBnHL with 20 dB increments to study the response threshold. The ground and positive electrodes were positioned on the forehead and the negative on the mastoid or on the zygomatic arc (near the ears), and the impedance was < 5kohm. The window used to study the latencies was 20ms, the stimulus presentation rate was 49.1/s; and 2000 responses were collected. The stimulus was supplied by the EAR 3A insertion phone. The polarity used was alternated. We used 30Hz high pass and 1500Hz low pass filters. The amplifier's rejection level was of 10± 25uV^8^.

For the BAEP (*Tone Burst*) the initial intensity was 80 dBnHL, with 20dB increments or decrements used to study the threshold. The electrodes were positioned as were the click ASSBA. The window for latency study was 25ms, and the stimulus presentation rate was 37/s; and 2000 responses were collected. The stimulus was supplied by the EAR 3A insertion phones. The polarity used was rarefaction. We used filters for 1k, 2k and 4kHz (100-3,000Hz) and for *Tone burst of* 500Hz (30-3000H)z^9^, ^10^.

For ASSBA, the placement of the electrodes and the earphones were the same as those for BAEP. The acoustic signal provided was made up by carrier frequencies of 500, 1k, 2k e 4kHz, respectively modulated in the amplitudes of 75, 85, 93, 101Hz on the left and 79, 87, 95, 103Hz on the right. The parameters used were a maximum of 400 responses analyzed at every 20 collections. The filters used were 70Hz and 110Hz.

The initial intensity was 60dBSPL in the multifrequency dichotic mode, and the increase or decrease in intensity depended on having a response. When no response was recorded, the ears were monaurally stimulated and the frequencies were presented separately.

**Chart 1:**
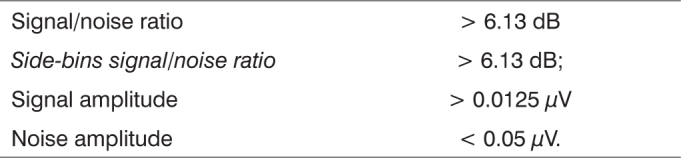
Values of the relations which were analyzed using the equipment's statistical method.

The data was submitted to the *Fast Fourrier Transform* analysis and angular analysis at every 20 collections, and p<0.05 was used as level of significance. We considered valid the frequency peaks corresponding to the modulation frequencies which were statistically higher than the noise level - we used the statistical method of the instrument, as depicted on Chart 1, below.

The maximum intensity supplied was 117dBSPL. During the procedure, if there were no responses recorded in the steady state register, we considered it a lack of response and the test was ended.

### Statistics

In the study of the correlation between the minimum response level at the ASSBA and the responses in the remaining tests, we calculated the Pearson's correlation coefficient^11^. This coefficient varies between -1 and 1, and values near zero indicate no correlation between the variables.

The agreement between the degree of hearing loss obtained from the ASSBA with the one obtained from the threshold prediction rule based on the acoustic reflex was assessed by means of the Kappa weighed statistical analysis^12^. The values of this coefficient can vary between -1 and 1. Values equal to or lower than 0.4 indicate poor agreement; between 0.41 and 0.6 moderate agreement; between 0.61 and 0.80 strong agreement; and from 0.81 to 1 indicates almost perfect agreement.

## RESULTS

The complete protocol was applied to 23 children 12 (52%) aged between 1 and 3 years, 9 (39%) were between 4 and 5 years and 2 children (9%) were between 6 and 7 years. Of these, 15 (65%) were females and 8 (35%) were males. Values for the descriptive statistical values for age are in years and are depicted on Table 1.

**Table 1:** Values from descriptive statistics for the age (years)

N	Mean	Standard Deviation	Minimum	Median	Maximum
23	3,4	1,6	1	3	7

We notice that most of the children have the same level of hearing loss in both ears. Only 1 child (4.4%) had profound hearing loss in the right ear and severe in the left; and 2 (8.7%) had severe hearing loss in the right ear and profound in the left, as depicted on Table 2.

**Table 2:** Distribution of joint, marginal percentages and frequencies of the degree of hearing loss on the right and left ears

			Left ear		
Right ear	Mild	Moderate	Severe	Profound	Total
Mild	1 (4,4%)				1 (4,4%)
Moderate		5 (21,7%)			5 (21,7%)
Severe			3 (13,0%)	2 (8,7%)	5 (21,7%)
Profound			1 (4,4%)	11(47,8%)	12(52,2%)

Total	1 (4,4%)	5 (21,7%)	4 (17,4%)	13(56,5%)	23(100%)

The results depicted on Table 3 (p-value) show that there is a significant correlation between audiometry and the ASSBA in the four frequencies considered, both in the right and the left ears. The same happened in the analysis of the correlation between the tone burst BAEP and the ASSBA. We notice that there was a significant correlation between the click BAEP and the ASSBA only in the 2k and 4kHz frequencies.

**Table 3:** Values from the Pearson's correlation coefficient (R) between ASSBA, Audiometry, click and tone burst BAEP per frequency and ear.

Frequency (kHz)	ASSBA and Audiometry		ASSBA and click BAEP		ASSBA and BAEP Tone Burst	
	Right	Left	Right	Left	Right	Left
	R	R	R	R	R	R
0.5	0,87	0,83	0,23	0,43	0,90	0,93
1	0,93	0,82	0,37	0,57	0,92	0,84
2	0,74	0,70	0,87	0,87	0,73	0,80
4	0,85	0,81	0,89	0,83	0,80	0,76

There was a moderate agreement between the hearing loss degree classifications and the ASSBA and the rule of predicting the degree of hearing loss based on the acoustic reflex - because there was an error in the degree of hearing loss (Tables 4 and 5).

**Table 4:** Frequencies and percentages of the hearing loss level based on the threshold estimation rule from the acoustic reflex and the ASSBA - Right ear.

		PEAEE		
	Mild or Moderate	Severe	Profound	Total
Severe	5 (21,7%)	2 (8,7%)	4 (17,4%)	11 (47,8%)
Profound			12 (52,2%)	12 (52,2%)

Total	5 (21,7%)	2 (8,7%)	16 (69,6%)	23 (100%)

Kappa = 0.45; standard deviation = 0.09

**Table 5:** Frequencies and percentages of the hearing loss level based on the threshold estimation rule from the acoustic reflex and the ASSBA - Left ear.

		PEAEE		
	Mild or Moderate	Severe	Profound	Total
Mild or Moderate	1 (4,3%)			1 (4,3%)
Severe	5 (21,7%)	2 (8,7%)	2 (8,7%)	9 (39,1%)
Profound			13 (56,5%)	13 (56,5%)

Total	6 (26,1%)	2 (8,7%)	15 (65,2%)	23 (100%)

Kappa = 0.60; standard deviation = 0.10

## DISCUSSION

In the analysis of the correlation between ASSBA and audiometry, seen on Table 3, we can notice that there was a significant correlation on the four frequencies considered to the right and the left. Other authors found a significant correlation for these procedures and suggested the use of ASSBA in clinical practice. A proper agreement between ASSBA and behavioral audiometry reinforces the possibility of its application in children who did not undergo VRA, favoring the diagnosis and the hearing rehabilitation as early as possible^13^, ^14^, ^15^, ^16^, ^17^, ^18^, ^19^, ^20^, ^21^.

The results obtained showed that there was a significant correlation between the ASSBA and the click BAEP; however, only for the 2k and 4kHz frequencies.

We also observed a strong correlation in the literature between the ASSBA threshold in 2kHz and the click BAEP (0.96) and between the 2k and 4kHz mean with the click (0.97)^22^. Recent studies have shown a good correlation between ASSBA and click BAEP (0.63 - 0.70), being better for the 1kHz frequency (0.70)^23^.

Click BAEP had already been correlated with behavioral assessment and results have shown correlations with the 2k and 4kHz frequencies. The click BAEP is usually the first measure applied in the audiological evaluation of children when the behavioral auditory responses are not successfully obtained. The click is not capable enough of estimating the threshold by specific frequency within the 500 to 4kHz spectrum^23^.

The click BAEP enables one to estimate the hearing loss degree, for a proposal of initial intervention, not providing details - as information per specific frequency. One of the main limitations of the click BAEP is the lack of a specific frequency. BAEP is highly dependent on neural synchrony. ASSBA overcomes some of the BAEP limitations, since it is a response evoked by a pure tone modulated in frequency and amplitude. It is common for the examiner to decide on the presence or absence of BAEP based on wave morphology under strong intensity^15^.

The discrepancy between click BAEP and ASSBA found in the present study was clear in the cases of hearing loss with descending configuration, since the click BAEP was absent and the ASSBA showed a preservation of the auditory threshold for the frequencies of 500 and 1kHz; then, we can consider that the click BAEP underestimated the hearing in children^15^. The prescription of amplification technological characteristics, referral for evaluation in a cochlear implant program and hearing rehabilitation are all based on the responses obtained.

The values of the correlation coefficients between Tone Burst BAEP and ASSBA varied between 0.73 and 0.93 for the frequencies of 0.5k and 4kHz. The results are in agreement with the ones already presented in the literature, since studies have shown a 0.86 correlation between the two tests for the frequency of 0.5kHz^23^ as well as for 0.25k and 0.5kHz we obtained the values of 0.9 and 0.79^17^. One recent study indicated that the correlation coefficient values were: 0.77; 0.60; 0.66 and 0.50 between 0.5k and 4kHz^22^.

Although the literature challenges the difficulty in reproducing the 500Hz frequency of the Tone Burst BAEP, in the present paper, the correlation with the AASSBA was 0.90 on the right side and 0.93 on the left side. Some authors described the morphology of the waves found as poor and which in many cases only wave V could be detected. Each change to the wave shape makes it difficult to identify and interpret the response^15^, ^24^.

Moreover, they reported that the wave shape for the low frequency stimulus tends to be less differentiated and more difficult to identify its click. The output limitation for low frequencies can then limit its use in the clinical practice as a protocol. They suggested that the ASSBA is an alternative because it used the continuous stimulus^22^.

There was a moderate agreement between the ASSBA classification and the hearing threshold estimate done based on the acoustic reflex.

On Tables 4 and 5 one can notice that 5 children (21.7%) had mild right and left side hearing loss according to the ASSBA, nonetheless, the rule for estimating the auditory threshold based on the acoustic reflex suggested that these children had severe hearing loss on the right and left. In the cases of profound hearing loss, 4 children (17.4%) on the right and 2 (8.7%) on the left were classified as having severe loss.

Although we have errors with the rule, it is worth mentioning that in no case the rule suggested normal hearing, since all the children assessed had sensorineural hearing loss. The most severe error may happen when the rule estimates the hearing to be normal in cases of severe hearing loss. A moderate error happens when the rule suggests normal hearing and there is moderate hearing loss; or when there is a severe hearing loss and the rule suggests moderate hearing loss. The errors in the hearing loss degree estimates have already been discussed in other studies^5^, ^25^, ^26^, ^27^, ^28^.

As we compare the values of pure tones in the study of acoustic reflexes with broad band noise, it was possible to notice that in 100% of cases the broad band noise threshold was either high or absent, suggesting hearing loss. When hearing is normal, according to summation, the broad band noise threshold is less intense when compared to pure tone^4^.

The analysis of the relation between the thresholds for pure tone and for broad band noise (white noise) indicated the difference in the auditory pathway behavior after the stimulus and this analysis could add information to the children audiological diagnoses; however, the auditory threshold estimation rule based on the reflex could not have been used alone^4^.

## CONCLUSÃO

ASSBA was significantly correlated with the audiometry (VRA and ludic), with the click BAEP for 2k and 4kHz and with the *tone burst* in all the frequencies, bilaterally.

The rule used to estimate the hearing loss can help and add information used to confirm the hearing loss, but it must not be used alone.
